# Mapping and candidate gene analysis of clustered bud on the main inflorescence in *Brassica napus* L.

**DOI:** 10.1186/s12870-023-04355-z

**Published:** 2023-07-04

**Authors:** Wen Yin Zheng, Zhe Yi Zhu, Abdul Sami, Meng Yuan Sun, Yong Li, Jian Hu, Xing Zhi Qian, Jin Xu Ma, Mei Qi Wang, Yan Yu, Fu Gui Zhang, Ke Jin Zhou, Zong He Zhu

**Affiliations:** 1 College of Agronomy, Anhui Agricultural University, Hefei, China; 2 National Key Laboratory of Wheat and Maize Crop Science, College of Agronomy, Henan Agricultural University, Zhengzhou, China

**Keywords:** *Brassica napus*, Population segregation analysis, Flower bud clusters, Endogenous hormones, Transcriptome analysis

## Abstract

Breeding rapeseed varieties with more main inflorescence siliques is an idea for developing rapeseed varieties that are suitable for light and simplified cultivation. The *Brassica napus* exhibited cluster bud of the main inflorescence (*Bnclib*) gene. At the fruiting stage, the main inflorescence had more siliques, higher density, and more main inflorescences. Moreover, the top of the main inflorescence bifurcated. Genetic analysis showed that the separation ratio between *Bnclib* and the wild type in the F_2_ generation was 3:1, which indicated that the trait was a single-gene-dominant inheritance. Among the 24 candidate genes, only one gene, BnaA03g53930D, showed differential expression between the groups (False discovery rate, FDR ≤ 0.05, |log2FC|≤ 1). qPCR verification of the BnaA03g53930D gene between Huyou 17 and its Bnclib near-isogenic line showed that BnaA03g53930D was significantly differentially expressed in the stem tissue of Huyou 17 and its Bnclib near-isogenic line (*Bnclib* NIL). The determination of gibberellin (GA), brassinolide (BR), cytokinin (CTK), jasmonic acid (JA), growth hormone (IAA), and strigolactone (SL) content in the shoot apex of Huyou 17 by *Bnclib* NIL and wild type showed that all six hormones significantly differed between the *Bnclib* NIL and Huyou 17. It is necessary to conduct further research on the interactions between JA and the other five hormones and the main inflorescence bud clustering in *B. napus*.

**Supplementary Information:**

The online version contains supplementary material available at 10.1186/s12870-023-04355-z.

## Background

Rapeseed (*Brassica napus* L.), a member of the Brassica family, is one of the most important oil crops worldwide and the largest oil crop in China (*Brassica napus* L.) [1]. The rape yield is influenced by the number of siliques present in the main inflorescence, particularly under high-density planting conditions. Song et al. [2] developed 15 recombinant inbred lines and connected traits, including the number of effective branches, number of seeds per silique, height of the first branch, number of siliques in the main inflorescence, effective length of the main inflorescence, and length of the silique. Following this investigation and genome-wide association analysis, it was discovered that the aforementioned traits showed phenotypic variation and that the majority of the traits were significantly correlated. Significant positive correlations were found between plant height and primary branch height, main inflorescence effective length, number of main inflorescence siliques, and silique length. According to studies conducted both domestically and abroad, numerous loci control the majority of features associated with the number of main inflorescent siliques [1].

Khan et al. [3]’s genome-wide association research identified 312 significant quantitative trait loci and 42 candidate genes in 521 rapeseed samples. Li et al. [4] identified six QTLs associated with the effective silique number of the main inflorescence and postulated that the candidate gene, BnaC05g32840D, for the number of effective siliques in the main inflorescence of rapeseed. They used 190 inbred lines of the DH-7–9 DH-G-42 hybrid as source material. Using the SSR molecular marker approach, Wolko et al. [5] conducted association analyses on eight key agronomic parameters of 63 *Brassica napus* populations and screened two markers that showed exceptionally strong correlations with the number of siliques. Additionally, other studies have indicated that there is only one dominant pair of genes that controls the main inflorescence-related traits. By combining numerous inflorescences and regular inflorescences as parents and using molecular marker technologies, Zong et al. [6] constructed a preliminary genetic linkage map and performed phenotypic identification of F_1_, RF1, and isolated populations. Near-isogenic lines have been extensively used in recent years to assist with gene mapping. Song et al. [7] identified 31 transcripts and 6 proteins related to maize photoperiod flowering by comparing transcriptomic and proteomic data. They discovered that the candidate gene, ZmCCT, is a homolog of the rice Ghd7 gene. They used a photoperiod-insensitive maize inbred line and its near-isogenotype photoperiod-sensitive line as source materials. The SP chromosomal segment between the TGS0308 marker and the C2 At5g49480 marker on tomato chromosome 1 was discovered by Nakano et al. [8] to both decreases and lengthen the tomato germination period at 15 °C and 25 °C. However, the chromosome 4 substitution fragment between SSR603 and TGS0411 had no effect on seed germination.

Endogenous hormones significantly influence the regulation of plant meristem development. Numerous hormones, including gibberellin (GA), brassinolide (BR), cytokinin (CTK), jasmonic acid (JA), growth hormone (IAA), and strigolactone (SL), are involved in several regulatory pathways that govern SAM and influence plant growth [9]. During plant growth, GA, BR, SL, and CTK control seed germination, organ elongation, the change from vegetative to reproductive growth, and flower, seed, and fruit development [10–12]. The leaf hormone, gibberellin, controls the induction of SAM floral organs, and research has shown that gibberellin can control flowering time [13]. IAA dynamically controls SAM growth and affects secondary structure formation in plants. Decreased IAA concentration affects cells in the surrounding area. JA, IAA, and GA play crucial roles in the growth and development of flowers, stems, leaves, and other organs in higher plants. JA and SL have been found in studies to limit plant development and induce leaf senescence [11–13]. In stressed plants, JA mediates signaling pathways that assist in defense and disease resistance [12]. In this study, an F2 segregated population of the corresponding combination was developed to identify and investigate the clustering traits of the main inflorescences and flower buds of rapeseed. Phenotypic and statistical analyses were performed to explore the genetic foundations of the main inflorescence flower bud cluster traits. The main inflorescence flower bud clustering trait of *Brassica napus* was clarified genetically using the (BSA) whole-genome resequencing approach and preliminary location of the major genes that influence it [14]. On this basis, candidate genes were predicted using bioinformatics analysis to locate the region.

## Material and methods

### Test material

Four parents were used in the experiment, including two inflorescence cluster flower bud lines, 12R1402 and 16R480 (a derived variety of 12R1402) and two wild-type lines, Huyou 17 and 7P71.

### Genetic analysis of Bnclib and construction of the mapping population

P1, P2, F1, and F2 of Combination I (12R1402 × Huyou 17) and Combination II (16R480 × 7P71) were planted in the Nongcui Garden of Anhui Agricultural University in fall, 2018. To generate the mutant and wild-type mixed pools, 20 individual plants from P_1_ and P_2_ of the two combinations, 20 individual plants of the main inflorescence clustered flower buds, and 20 individual plants of the wild type from the F_2_ segregated population in the two combinations were selected. In the Nongcui Garden of Anhui Agricultural University, P_1_, P_2_, and F_1_, F_2_ of Combinations I (12R1402 × Huyou 17) and II (16R480 × 7P71) were planted in the autumn of 2019. Each F_2_ individual plant's clustered flower buds and inflorescence angle was examined during the budding and pod-setting stages. Prior to initial flowering, the visual method was used to investigate and record the main inflorescence cluster bud phenotypes of P_1_, P_2_, F_1_, and F_2_ of combination I (12R1402 × Huyou 17) and combination II (12R480 × 7P71), and to investigate and record the main inflorescence angle and role at the final flowering stage. The main inflorescence-clustered flower bud phenotype was identified by main branch pod density, multiple main branches, and the top branch of the main branches when the investigation results from the two periods were combined. The actual segregation ratio of the clustered flower bud phenotype of the F_2_ main inflorescence was compared with the theoretically predicted ratio using the fitness test in the chi-square test.

### Paraffin sections of Bnclib mutant and wild-type flower buds

The experiment was performed according to the method described by Hadfi et al. [15]. Centrifuge tubes (10 mL) were filled with 9 mL of FAA fixative. Eight field samples were collected from three wild-type plants, three 12R1402 mutant plants, and two 16R480 mutant plants. A blade of approximately 1 cm × 1 cm was used to remove the buds of the main inflorescence and placed in a centrifuge tube to ensure that the samples were soaked in the FAA fixative. The flower buds were transferred to 70% ethanol, excess parts were removed, and the top circular structure was exposed. The samples were treated with 50%, 30%, and 10% ethanol solutions and transferred to distilled water. They were then stained for 96 h with 1% eosin and hematoxylin dilution solution, rinsed three times with distilled water after complete staining, and then gradually dehydrated for three hours. Xylene, anhydrous ethanol, and pure xylene were graded and mixed at ratios of 1:4, 1:2, and 2:1 for two hours each after the samples had been dehydration. Paraffin infiltration was performed and embedding was carried out, paraffin was trimmed, and the sample was horizontally sliced with a thickness of 8 μm. Images were captured using a microscope (Nikon, Tokyo, Japan). Scanning was carried out with the use of a Pannoramic 250/MIDI scanner (3D HISTECH).

### Construction and genotyping of extreme pools

We extracted DNA from 20 individual wild-type plants (BL), 20 mutant plants (BM), and 20 clustered main inflorescence parents (PM) from combinations I and II, as well as from the F_2_ population of the two combinations. To construct a mixed pool of mutant and wild-type traits, DNA from 20 flower bud cluster inflorescence F_2_ individual plants and 20 wild-type F_2_ individual plants were mixed in equal amounts. We used a hole punch to remove three 1.5 cm-diameter leaf fragments from each plant in the field based on its phenotype (20 plants from BL, BM, PL, and PM). Initially, samples were submerged in liquid nitrogen and later stored in a freezer at -80 °C.

### DNA extraction and PCR amplification

DNA was extracted as previously described [16]. Samples were ground in liquid nitrogen. Approximately 150 mg of young leaves was taken, and 6 μL of ribonuclease A (RNase A) and 500μL of LP1 buffer were added. The treated samples were placed in a grinder for 80 s, vortexed for 60 s, and then kept for 8–10 min at room temperature. Centrifuge tubes were taken and 150 μL of LP2 buffer was added, they were shaken for 60 s, and then centrifuged at 4 °C and 10,000 rpm for 8 min, and supernatant was taken. Then, 1000 μL of LP3 buffer containing 100% ethanol was added, shaken evenly, and transferred to the adsorption column. Centrifugation was carried out for 60 s. 500 μL of GW2 buffer containing anhydrous ethanol was added to adsorption column and centrifuged at 10,000 rpm for 3 min. 100 μL of ddH2O was added to dilute samples, and centrifuged at 12,000 rpm in a 4 °C low-temperature for 60 s, and then kept at -20 °C for future use. 10 μL system configuration: 0.1 μL taq synthase, 0.5 μL forward primer, 0.5 μL reverse primer, 0.8 μL 2.5 mM dNTP, 1.0 μL DNA template, 1.0 μL 1xbuffer buffer solution, 6.1 μL ddH_2_O trim, and 20 μL of paraffin oil for future use. The PCR amplification program used is listed in Table S13.

### Bioinformatics analysis of Clean Reads

High-throughput sequencing of libraries that met the standards after quality inspection was performed using an Illumina HiSeq platform. The original paired-end sequences (reads) obtained by sequencing were subjected to Base Calling (base calling) using Illumina Casava version 1.8. Quality was assessed using the base quality distribution map and base-type distribution map. The resulting clean read files were stored in FASTQ format. The reference gene information of version Brassicanapus_v4.1 from http://www.genoscope.cns.fr/brassicanapus/data/ was compared with Clean Reads to obtain alignment efficiency, insert analysis, and genome coverage data charts, such as degree and coverage depth. Samtools software was used to separate the overlapping sequencing sites, and single nucleotide polymorphisms (SNP) were detected after correcting the base quality value (SNP) and small indels (Small InDel) [17]. The detected SNP and small InDel information were combined to perform association analysis, gene screening, mapping of associated regions, and SNP annotation to predict the impact of variation. SnpEff software was used to annotate variants (SNP, Small InDel), predict the effect of variants on NP, and analyze the statistical table of annotation results. GATK software was used to detect indels of samples to obtain InDel statistical results table between samples, and breakdancer software was used to detect the results. The insert size and variance of the sequencing data library were calculated, and the SV detection of the comparison results was used to obtain a statistical table of the number of SVs of various types. GO, KEGG, and COG annotations were performed to analyze the function of genotypes.

### Screening of molecular markers closely linked to Bnclib and construction of Bnclib near-isogenic line

**Fig. 1:**
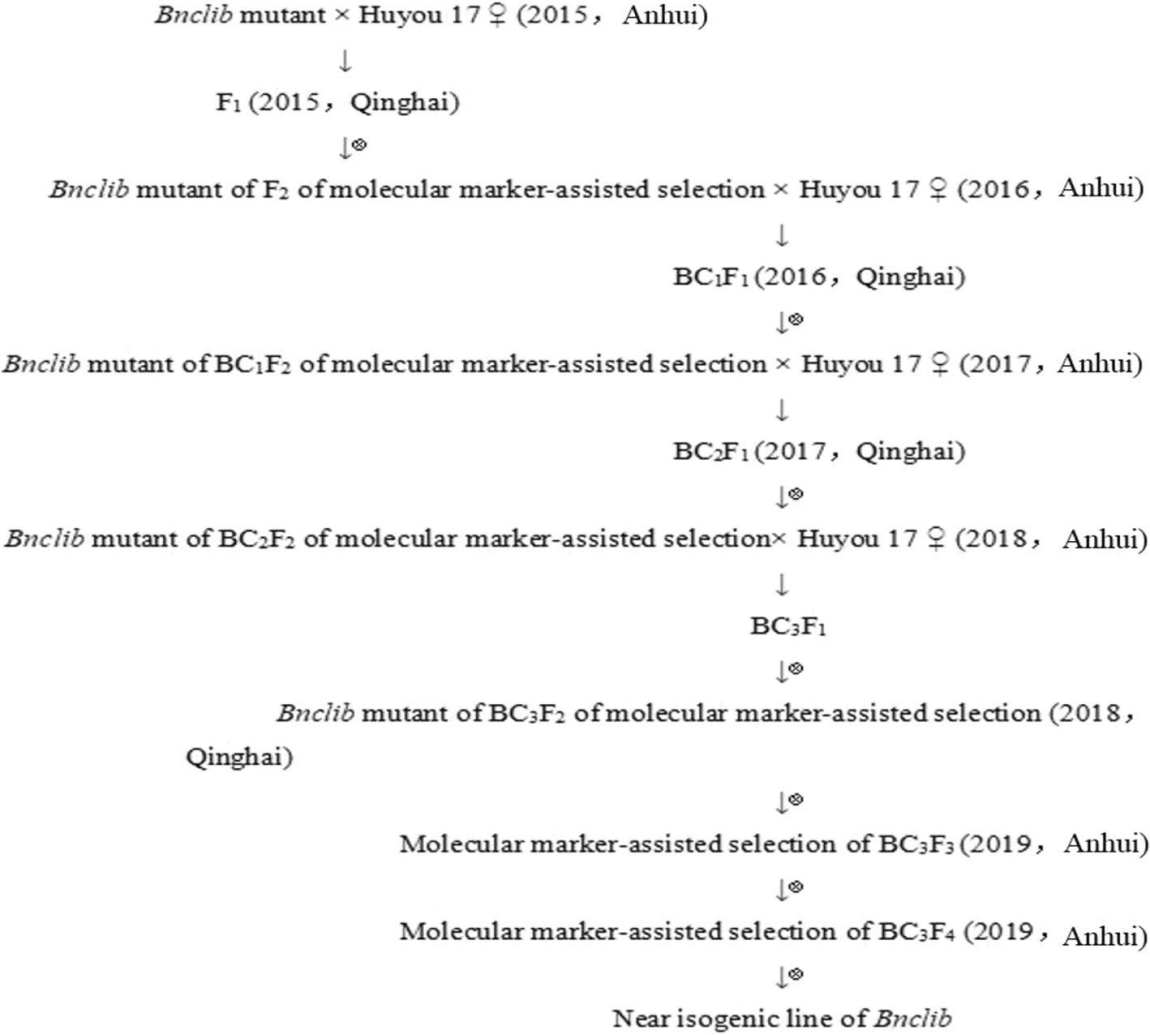
Construction process of the Near-isogenic line of *Bnclib*

Based on the findings of whole-genome re-sequencing and the Brassica database (BRAD), 1292 pairs of SSR primers and 131 pairs of InDel primers were designed for screening of molecular markers that were closely associated with Bnclib. 12R1402 was backcrossed with Huyou 17, and a Bnclib near-isogenic line was constructed by screening molecular markers closely linked to Bnclib for transcriptome analysis. Population segregation analysis was performed after flowering, and the Bnclib near-isogenic line was constructed in the experimental fields of Hefei and Haidong City of the Anhui Agricultural University from 2015 to 2020 (Fig. 1).

### Transcriptome analysis

Twenty stem samples from the 12R1402, Bnclib near-isogenic, and Huyou-17 lines were randomly selected at the flower bud differentiation stage. Total RNA was extracted and eukaryotic mRNA was enhanced using magnetic beads and oligo (dT). 1.5% agarose gel electrophoresis was performed [17]. The mRNA was randomly broken into short sequences, reverse-transcribed into cDNA, and second-strand cDNA was synthesized. Following purification, the poly (A)-tailed, end-repaired, and ligated double-stranded cDNA was attached to sequencing adapters [18]. PCR amplification and sequencing were performed on an Illumina HiSeq 2500 platform (Illumina Inc., San Diego, CA, USA) by Kidio Biotechnology Co., Ltd. (Guangzhou, China).

FPKM (Fragments per kilobase of transcript per million mapped fragments) was used to measure the gene expression levels. During the detection of differentially expressed genes, log2 (Fold Change) |≥ 1 and FDR < 0.05 were used as screening criteria. Fold Change represents the ratio of expression levels between two samples (groups). The False Discovery Rate (FDR) was calculated by accounting for the *p*-value of significant differences. Gene differential expression (DEGs) with higher levels of expression in Bnclib than in the wild type were labeled as up-regulated, whereas DEGs with the opposite relationship were labeled as down-regulated.

### Detection of GA, CTK, IAA, SLs, JA, BR content in stem

Plant hormone levels were determined as described previously [19–25]. A total of 60 samples, 30 samples of 1 cm × 1 cm size from the shoot tips of Bncilb and wild type plants, were collected from the field. Five samples of each hormone were collected from the shoot tips of Bncilb and wild type plants. Jiangsu Yutong Biotechnology Co., Ltd. measured the hormone content of the samples (Jiangsu, China). Ten microliters of the sample and 50 μL of the standard were added to the corresponding wells after centrifuging the plant extract at 1000 rpm for 10 min. Add 100 μL of HRP-labeled detection antibody to the well, and this was then heated in a water bath at 37 °C for one hour. 50 μL of substrate A and 50 μL of substrate B was added to each well, and then incubated at 37 °C in the dark for 15 min. The TMB substrate developed color, and the color depth was related to the measured hormone content. The darker the color, the higher the hormone contents. 50 μL of stop solution was added to the well, a microplate reader was used to measure the OD value at 450 nm, a standard linear regression curve was made according to the OD value, and the sample concentration was calculated.

## Results

### Morphological observation and identification of Bnclib mutants

**Fig. 2:**
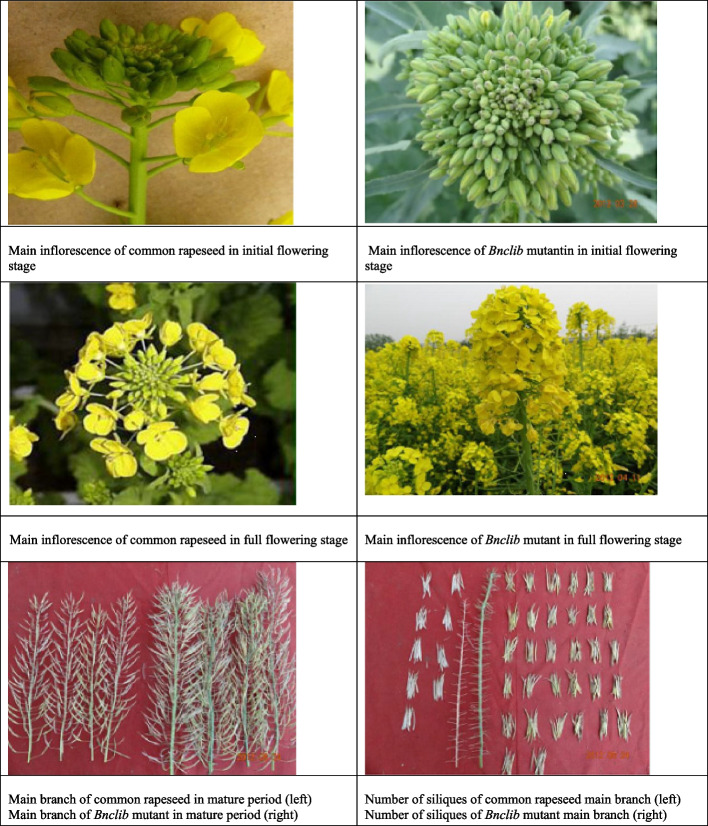
Flower bud of cluster main inflorescence and flower bud of wild type inflorescence. Morphological observation and identification of Bnclib mutants

**Fig. 3:**
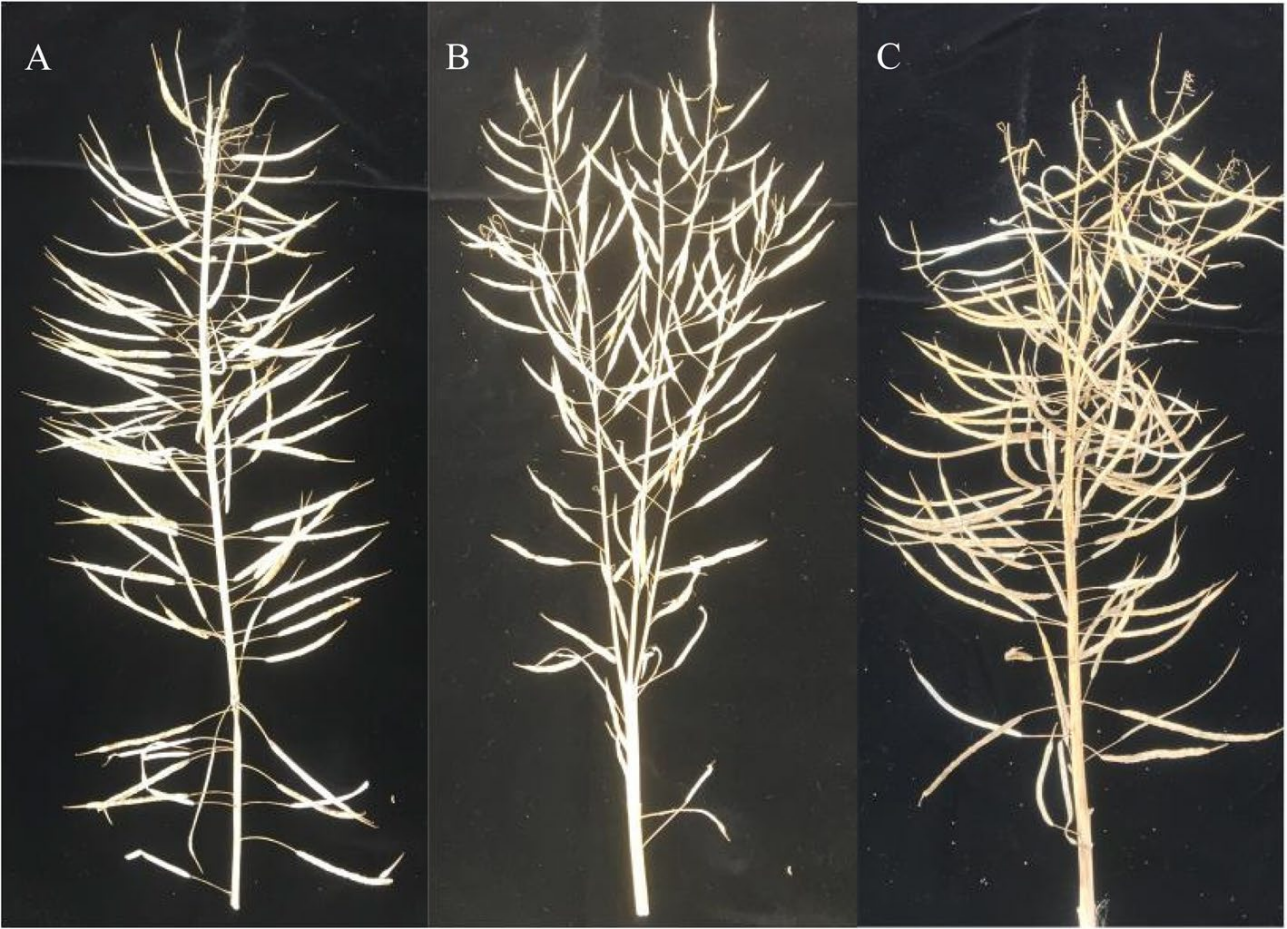
Types of clustered buds in the main inflorescence of the F_2_ population. **A** Type I; **B** Type II; **C** Type III

The main bud cluster material 12R1402 had 2–4 inflorescences at the top of the main inflorescences, with an average of three inflorescences in the clusters and increased number of flower buds. The average number of flower buds in the main inflorescences was (175 ± 25) (Fig. 2). Wild-type Rapeseed: The main inflorescence had only 1 growing point and the average number of flower buds in the main inflorescences was (75 ± 10) in the sprout stage. The phenotype of F_1_ includes some plants with cluster buds in a single main inflorescence, whereas most plants have 2–3 inflorescences at the top of the main inflorescence. Phenotypic identification and analysis of the F_2_ isolated populations showed that, in addition to a single plant with the same phenotype as the two parents, there were also intermediate traits. The primary inflorescences of F_2_ were divided into three types, which belonged to the type of clustered buds: type I had a single main inflorescence with cluster buds and no branch at the top (Fig. 3A), and the density and number of siliques were significantly higher than those of the wild type, type II had 2–4 main branches (Fig. 3B), and the density and number of siliques per main branch were the same as those of the wild type, and type III had one main branch, several branches at the top of the main branch, and a normal branch angle (Fig. 3C). The total number of siliques in the main branch was substantially higher than that in the WT. Conclusively, F2 main inflorescence type I accounted for 15%, type II accounted for 25%, and type III accounted for 60%.

### Results of Bnclib genetic analysis

Crosses I (12R1402 × Huyou 17) and II (16R480 × 7P71) were used to ascertain the genetic makeup of the major inflorescence-clustered flower buds of the Bnclib mutant. All plants in the F_1_ population of combinations I and II had Bnclib characteristics, and there was no trait segregation. However, the primary inflorescence bud cluster was less robust than the parent. A total of 288 plants in the F_2_ population of combination I developed clustered flower buds on their main inflorescences, whereas 82 plants did not. There were 243 plants in the F_2_ population of combination II, of which 177 had clustered main bud inflorescences and 66 did not. The results of the orthogonal and anticross analyses were consistent, and the Bnclib characteristics of the F_2_ generation and wild type followed Mendel's law of genetic separation with a 3:1 separation ratio, according to the chi-square test (Table S1). As a result, the *Brassica napus* L. main inflorescence bud cluster character, known as Bnclib, is a single gene-dominant character (cluster buds of the main inflorescence in *Brassica napus* L.).

### Paraffin section analysis of Bnclib mutant and wild-type flower buds

**Fig. 4:**
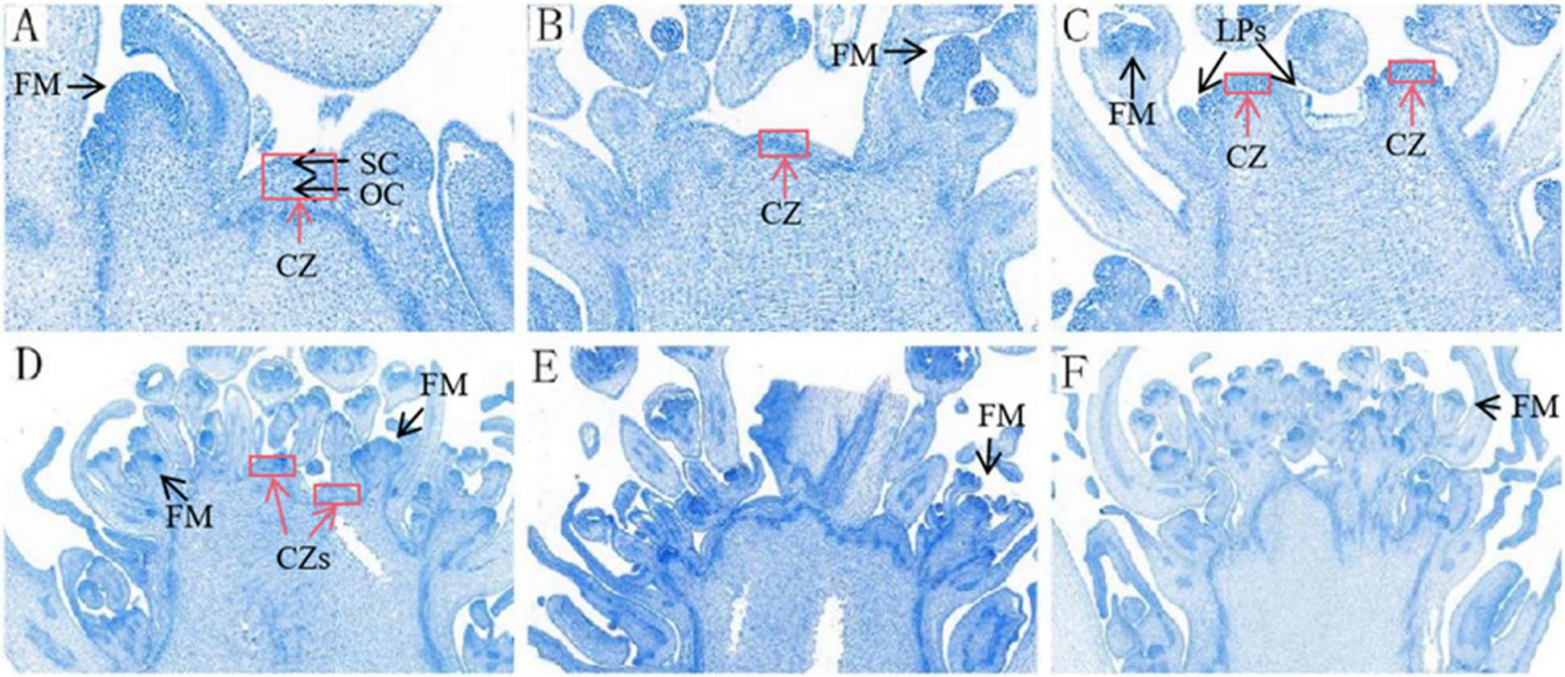
Microstructure of paraffin section of apical meristem. **A** Huyou 17 (wild-type); **B-C**: 16R480 (*Bnclib* mutants); **D-F** 12R1402 (*Bnclib* mutants) SC: Stem cells; OC: Organizing center; CZ: Central zone; LP: Leaf primordium; FM: Floral meristem. Paraffin section analysis of Bnclib mutant and wild-type flower buds

Phenotypic analysis revealed that at the bolting/moss extraction stage, the main cluster inflorescences of the Bnclib mutant were considerably different from those of the wild type. The Bnclib gene mutation was the cause of the phenotypic variation because the sowing time and field management practices of the mutant and wild type were the same. The main inflorescences of the mutant and wild-type plants were significantly different in terms of the number of flower buds and inflorescence shape. To explore the growth characteristics of the main inflorescences of the mutant and wild-type plants, samples were collected during the flower bud differentiation stage to observe the meristem structure. Observation of the paraffin sections revealed that the structure of the apical meristem of wild-type plants was loose, the central region (CZ) was obvious, and the formation of the flower apical meristem (FM) was reduced (Fig. 4A). The structure of the central region of the Bnclib mutant was not obvious, with multiple meristem differentiations and significantly increased flower bud numbers (Fig. 4B-F). The wild-type CZ structure was more evident, and the stem cell region (SC) and tissue center (OC) were clearly observed. The number of FM in the mutant was higher than that in the wild type. The FM is on the flank of the apical meristem (SAM), which is a specific meristem for the formation of flowers. An increase in the FM number leads to a cluster of flower buds in the main inflorescence of rapeseed. Some mutant plants had multiple SAMs (Fig. 4C, D), and the newly split SAMs continued to divide and differentiate into new intact meristems.

### Extreme population mixing pool construction and sequencing data analysis

A mixed pool of 20 individual plants (R01) with mutant traits and 20 wild-type plants (R02) was constructed from the F_2_ population in combination I (Table S2). The entire gene resequencing of the F2 population was performed to obtain original data. After filtering, 59.02 GB of data were obtained, and the Q30 of R01 and R02 were 93.30% and 93.40%, respectively (Table S2). The comparison efficiencies between the sample and reference genomes were 98.09% and 98.80%, respectively (Table S3). The average coverage depth was 32 × to 39x, and the genome coverage was 97.54%. A mixed pool was constructed for 20 main inflorescence cluster parents (20 strains) 16R480 (PL) in combination II, 0 wild-type parent 7P71 (PM), and 20 mutant single plants (BM) and wild-type single plants (BL) in F_2_ populations with obvious cluster characteristics of main inflorescence buds in the two combinations; whole gene resequencing of the two parents was carried out to obtain the original data (Table S4). After filtering, the valid sequence data for the PL, PM, BL, and BM samples reached 127.35 GB. The GC content was 37.37–37.53%, the lowest Q20 value was 95.99%, and the average was 96.22%; the lowest Q30 value was 89.13%, and the average was 89.62%. The lowest comparison rate of sample sequencing reads was 99.16%, and the average was 99.30%. The read comparison rate of paired-end sequencing sequences was 98.79%, and the average was 98.98% (Table S5). The paired-end sequencing sequences could be aligned to the reference genome, and the alignment rate of the fragments of suitable size was the lowest at 89.66%, and the average was 90.29% (Table S5). All of the above alignment efficiencies for BL, BM, PL, and PM were more than 99%, and the overall values were ideal. Furthermore, all samples contained sufficient data and the sequencing quality was acceptable. The GC content was similar and evenly distributed without separation. The comparison efficiency between the sequencing data and the reference genome of rapeseed was higher than 70%, and the overall data were ideal and could be used for subsequent variation detection and gene location of traits.

### Bnclib trait gene mapping

#### ΔSNP and ΔInDel analysis of combination I (12R1402 × Huyou 17) mixed pool

**Fig. 5:**
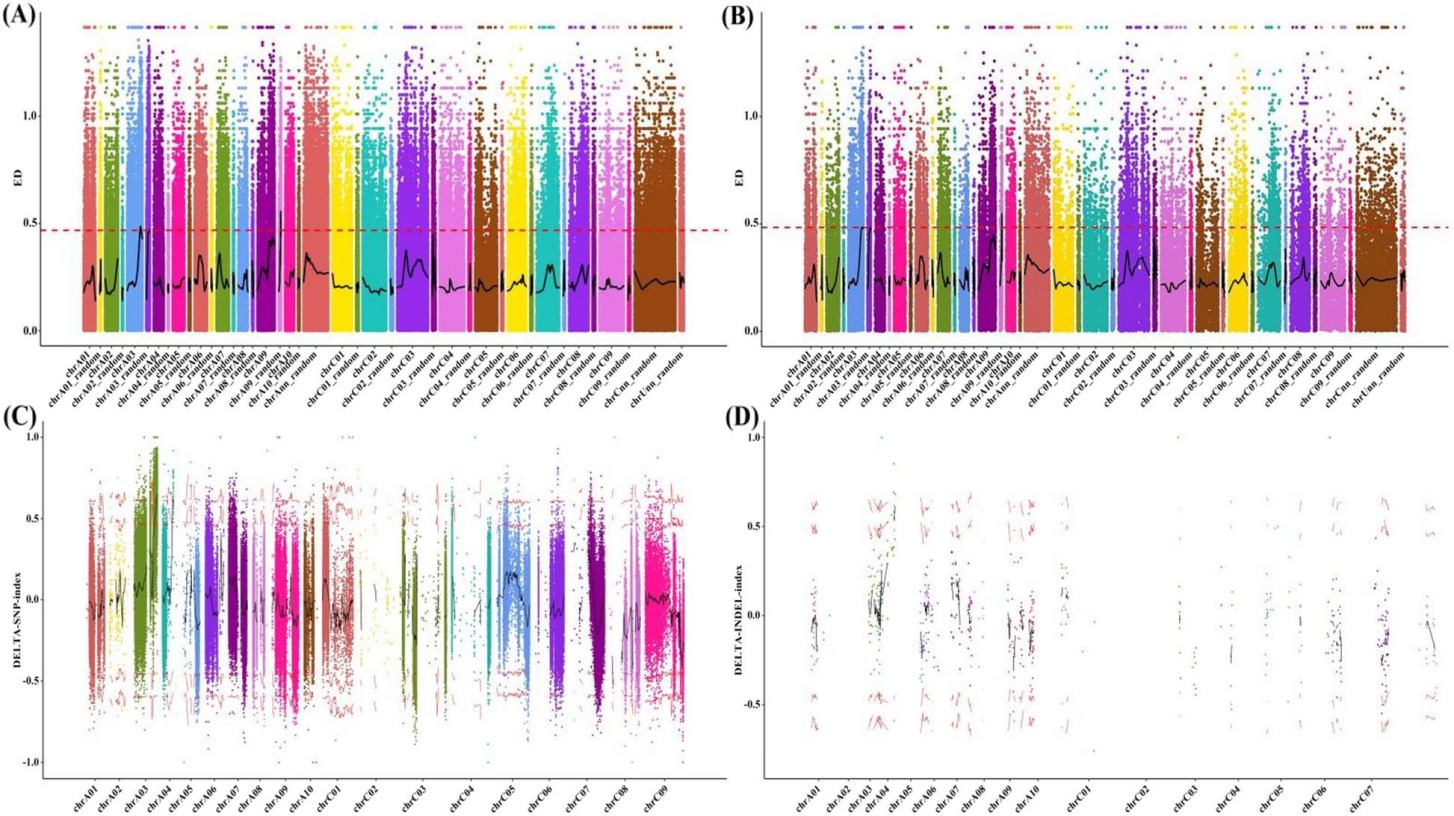
ΔSNP and ΔInDel analysis among mixed pools of combination I and II. **A** F_2_ population ΔSNP-index values across the genome in combination I. **B** F_2_ population ΔInDel-index values across the genome in combination I. **C** Distribution of F_2_ population ΔSNP-index values across the genome in combination II. **D** F_2_ population across the genome in combination II. Bnclib trait gene mapping

The ΔSNP-index correlation analysis was performed on the two mixed pools of the combination I F_2_ population. According to the localization data, four regions on *Brassica napus'* two chromosomes were found to have extremely significant peaks that exceeded the critical value level (Fig. 5A). This suggests that these regions may contain genes that regulate Bnclib variation in *Brassica napus*. On the rape genome, these four significant association intervals for genes with Bnclib variations were distributed as follows: chrA03 25.37–27.59 Mb, chrA03-random 4.41–4.64 Mb, chrA03-random 5.77–5.81 Mb, and chrA09-random 3.39–4.02 Mb (Fig. 5A, Table S6). The four significantly associated regions contained 357, 12, 2, and 136 identified genes. The two pools of the combination I F_2_ population were subjected to ΔInDel-index correlation analysis. These findings demonstrated that the rapeseed genome contained two areas that were distributed across *Brassica napus'* two chromosomes and had exceptionally significant peaks that exceeded the threshold level (Fig. 5B). The distribution of these two significant association intervals on the rape genome was chrA03 26.68–28.50 Mb and chrA09-random 3.31–3.89 Mb. There were 257 and 120 annotated genes in the two significantly associated regions. Four significant correlation intervals may contain genes that regulate Bnclib variation in *Brassica napus*, according to combined SNP and ΔInDel analyses between mixed pools of combination I, ChrA03 25.38–28.50 Mb, CHRA03-Random 4.41–4.64 Mb, CHRA03-Random 5.77–5.81 Mb, and CHRA09-Random 3.31–4.02 Mb (Fig. 5A, Table S7).

#### ΔSNP and ΔInDel analysis among mixed pools of combination II (16R480 × 7P71)

The ΔSNP-index correlation analysis of the two mixed pools in the combination II F_2_ population showed significant peaks in three regions of the two chromosomes exceeding the critical level (Fig. 5C). The distribution of these three significant association intervals on the rape genome was chrA03 28.30–29.69 Mb, CHRA03-random 2.30–6.02 Mb, and chrA04 12.77–13.81 Mb (Fig. 5C, Table S8). There were 98, 151, and 154 annotated genes in the three significantly associated regions.

According to the findings of the ΔInDel-index correlation analysis performed on the two pools of the combination II F_2_ population, only one region on one chromosome had a very significant peak that exceeded the critical level (Fig. 5D), and the distribution of the significant correlation interval in the genome was chrA03-random 4.97–5.76 Mb. There were 31 annotated genes identified in the significantly associated regions. SNP and InDel analysis of mixed pools in combination II revealed that chrA03 was 28.30–29.69 Mb, CHRA03–Random was 2.30–6.02 Mb, and chrA04 was 12.77–13.81 Mb, indicating that three significant correlation intervals may contain genes regulating Bnclib variation in *Brassica napus* (Fig. 5D, Table S9).

#### Candidate gene for Bnclib trait

The cluster characteristics of the main inflorescence buds of combinations I (12R1402 × Huyou 17) and II (16R480 × 7P71), based on BSA, and were derived from the common parent 12R1402. Common association regions (mainly SNP analysis) of the two combinations, based on the initial mapping of BSA, were selected as candidate Bnclib trait genes. The candidate regions were: (1) chrA03 28.30–28.50 Mb and (2) CHRA03-RANDOM 5.78–5.81 Mb, with 22 and 2 candidate genes, respectively (Table 1).

**Table 1:** Statistical table of candidate genes

Serial number	Candidate gene	Start	Termination
1	*BnaA03g53760D*	28304810	28306762
2	*BnaA03g53770D*	28306819	28309275
3	*BnaA03g53780D*	28313037	28315127
4	*BnaA03g53790D*	28332388	28335178
5	*BnaA03g53800D*	28355753	28359162
6	*BnaA03g53810D*	28363833	28364225
7	*BnaA03g53820D*	28369216	28372710
8	*BnaA03g53830D*	28390679	28393341
9	*BnaA03g53840D*	28401675	28404044
10	*BnaA03g53850D*	28404566	28405220
11	*BnaA03g53860D*	28425197	28427930
12	*BnaA03g53870D*	28441061	28441858
13	*BnaA03g53880D*	28444524	28444742
14	*BnaA03g53890D*	28454964	28455218
15	*BnaA03g53900D*	28456104	28456899
16	*BnaA03g53910D*	28459534	28460378
17	*BnaA03g53920D*	28464368	28466644
18	*BnaA03g53930D*	28473365	28475435
19	*BnaA03g53940D*	28476088	28478294
20	*BnaA03g53950D*	28479034	28479504
21	*BnaA03g53960D*	28485450	28486567
22	*BnaA03g53970D*	28488248	28490423
23	*BnaA03g60700D*	5786795	5787251
24	*BnaA03g60710D*	5787740	5788278

### Screening of markers linked to Bnclib and construction of near-isogenic lines

**Fig. 6:**
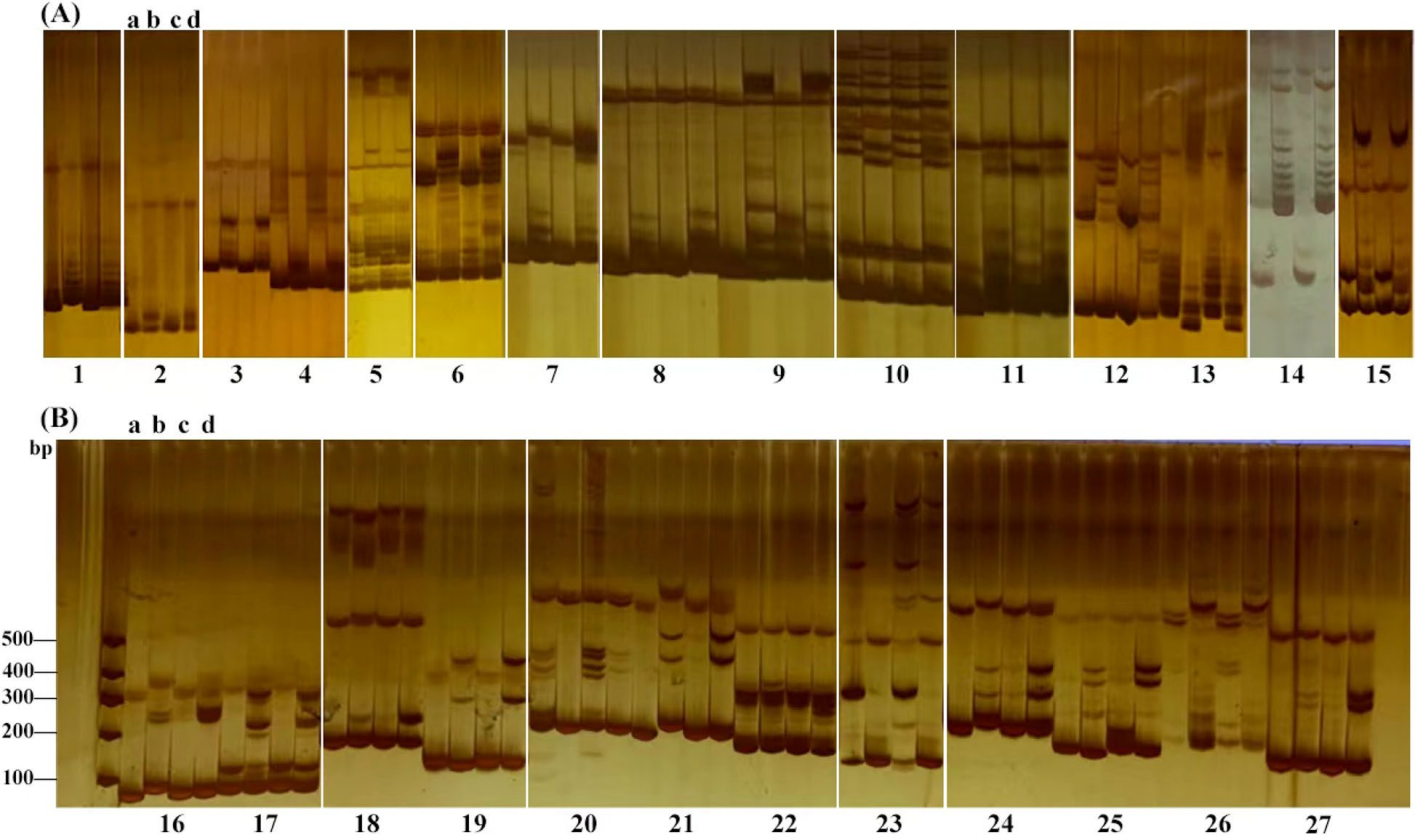
**A** Fifteen SSR markers revealed the polymorphisms among two parents and two bulks. **B** Twelve InDel markers revealed the polymorphisms among two parents and two bulks. **a** 12R1402; **b** Huyou 17; **c** *Bnclib* bulk; **d** Normal bulk. Screening of markers linked to Bnclib and construction of near-isogenic lines

In total, 131 pairs of InDel primers and 1292 pairs of SSR primers were developed for the candidate regions of Bnclib identified by BSA whole-genome resequencing analysis. The primers that exhibited polymorphisms in the main inflorescence cluster parent 12R1402 and the common main inflorescence parent Huyou 17 were screened from 1292 pairs of SSR and 131 pairs of InDel primers. Polymorphism markers between these two parents were marked on the F_2_ main inflorescence. To screen out the markers associated with the target main inflorescence cluster gene, the clustering pool (Bnclib bulk), and normal main inflorescence pool (normal bulk) were amplified. After rigorous screening, 12 pairs of InDel primers and 15 pairs of SSR primers (Fig. 6A-B, Fig. S2, Table S10, 11) were found to contain polymorphisms between the two gene pools and the parents.

**Fig. 7:**
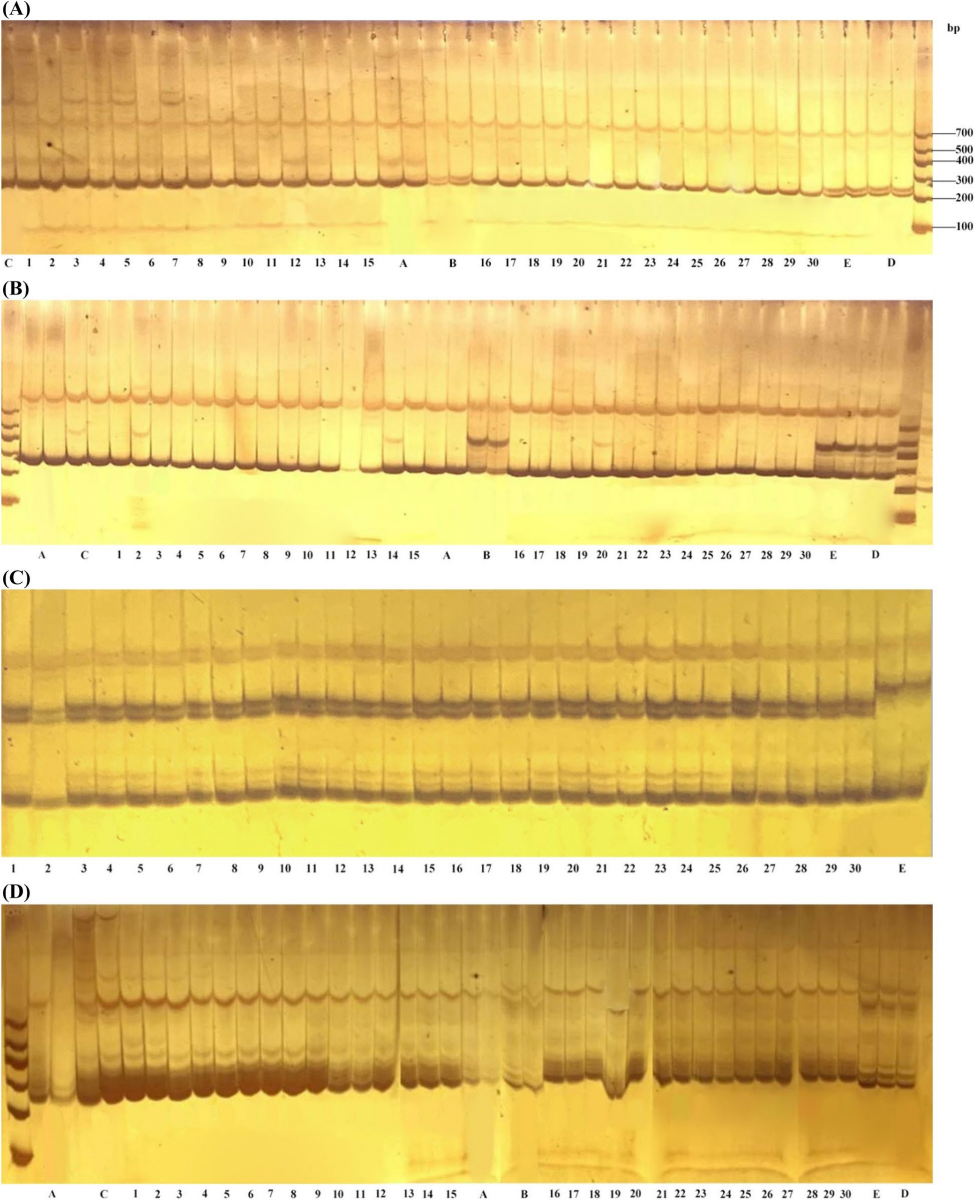
**A** Electrophoretic image of primer chrA03-2. **B** Electrophoretic image of primer chrA03-3. **C** Electrophoretic image of primer chrA03-6 (Plants No. 1–30 are NIL plants, plants indicated by the arrow are plants with normal bud of main inflorescence). **D** Electrophoretic image of primer chrA03-13

The homozygous dominant F_3_ without separation of the bud cluster main inflorescence and common main inflorescence was chosen for strict screening of 27 pairs of linkage markers, and the primers that appeared to be banded were eliminated. This was performed using the self-cross-progeny F_3_ of a single plant of the cluster’s main inflorescence isolated from F_2_. Four pairs of primers (Table S12) that were completely consistent with the band type of the main inflorescence cluster parent and showed polymorphism and a clear band of the common main inflorescence parent were selected to assist in the construction of the near-isogenic line of the main inflorescence cluster parent (Fig. 1). Prior to each backcross, 30 non-recurrent parental lines were chosen for amplification with four pairs of primers, and four near-isogenic lines with identical primers were chosen as non-recurrent parents and backcrossed with Huyou 17 (Fig. 7A-D, Fig. S3).

### Transcriptome analysis

The transcriptome analysis of the main inflorescence bud cluster material 12R1402, the common main inflorescence Huyou 17, and the main inflorescence bud cluster near-isogenic line constructed using Huyou 17 as the recurrent parent, generated differential expression data for 24 potential genes (Table 2). Transcriptome analysis revealed significant differences in the expression of 24 candidate genes between the main inflorescence bud cluster material 12R1402 and the common main inflorescence Huyou 17 in BnaA03g53750D, BnaA03g53920D, and BnaA03g53930D. BnaA03g53930D was the only common gene in the candidate region that exhibited a difference between groups in the transcriptome study between the common main inflorescence of Huyou 17 and the main inflorescence bud cluster near-isogenic lines. BnaA03g53930D is homologous to CYP81D11 reported in Arabidopsis, according to gene annotation information from NCBI (https://www.ncbi.nlm.nih.gov/), TAIR (https://www.arabidopsis.org/), and Genoscope. Functional annotation of homologous genes in Arabidopsis describes a cytochrome P450 gene that regulates JA synthesis. The relative expression levels of the gene BnaA03g53930D in the flower, stem tip, and leaf tissues of mutant and wild-type inflorescences were measured by qRT-PCR. The results showed that the BnaA03g53930D gene was significant in the stem tip tissues of mutant and wild-type plants, but not in flowers and leaves (Fig. S1).

**Table 2:** Differential expression of candidate genes

Candidate geneID	Huyou17 vs 12R1402		Huyou17 vs *Bnclib* NIL		
	log_2_(FC)	FDR	log_2_(FC)	FDR	Symbol
*BnaA03g53750D*	-8.714	0.045	-2.585	0.265	*HSFB1*
*BnaA03g53760D*	0.025	1.000	-0.432	0.483	-
*BnaA03g53770D*	0.111	0.496	0.071	0.865	*RNP1*
*BnaA03g53780D*	0.000	1.000	0.000	1.000	*PURA1*
*BnaA03g53790D*	0.000	1.000	0.000	1.000	*RPA1D*
*BnaA03g53800D*	1.000	0.872	2.644	0.078	*At5g67130*
*BnaA03g53810D*	0.000	1.000	0.000	1.000	-
*BnaA03g53820D*	-0.075	0.689	0.141	0.461	*NAPRT1*
*BnaA03g53830D*	-0.598	0.000	-0.219	0.125	*AP2*
*BnaA03g53840D*	-0.196	0.689	-1.148	0.001	*CBSX1*
*BnaA03g53850D*	0.835	0.000	-0.546	0.173	*RAP2-10*
*BnaA03g53860D*	2.763	0.159	0.193	1.000	-
*BnaA03g53870D*	0.000	1.000	0.000	1.000	*MYB44*
*BnaA03g53880D*	0.000	1.000	0.000	1.000	-
*BnaA03g53890D*	0.000	1.000	0.000	1.000	-
*BnaA03g53900D*	0.000	1.000	0.000	1.000	-
*BnaA03g53910D*	0.000	1.000	0.000	1.000	*PUB62*
*BnaA03g53920D*	-1.376	0.015	-1.230	0.062	*CYP81D11*
*BnaA03g53930D*	2.401	0.000	1.498	0.008	*CYP81D11*
*BnaA03g53940D*	0.272	0.658	0.152	1.000	*CYP81D11*
*BnaA03g53950D*	0.000	1.000	0.000	1.000	-
*BnaA03g53960D*	0.001	1.000	0.134	0.128	*AGP17*
*BnaA03g53970D*	0.000	1.000	0.000	1.000	*SRFR1*
*BnaA03g53980D*	-0.184	0.429	-0.047	1.000	*KAI2*
*BnaA03g60700D*	0.000	1.000	0.000	1.000	-
*BnaA03g60710D*	0.000	1.000	0.000	1.000	-

### Determination of endogenous hormones

**Fig. 8:**
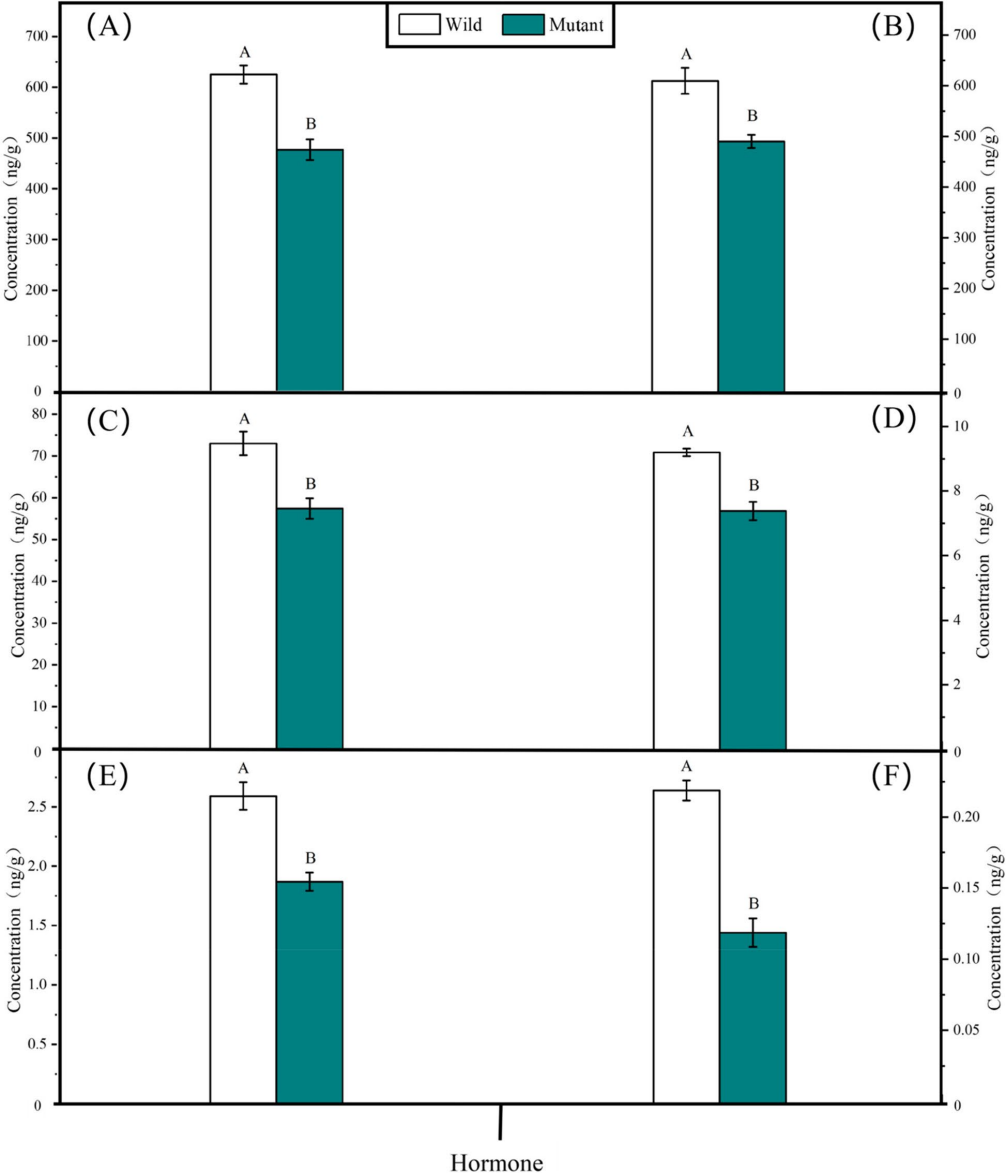
Concentration of hormones in wild-type and mutant plants. **A** GA; **B** CTK; **C** IAA; **D** SLs; **E** JA; **F** BR. Determination of endogenous hormones

GA, CTK, IAA, SLs, JA, and BR were detected in five samples of clustered mutant flower buds from the main inflorescence and five samples of wild-type flower buds (Fig. 8). The levels of the six hormones in the flower buds of the two groups were significantly different. The GA and CTK contents were the highest in the samples, both of which were above 600 ng/g for the wild type and above 450 ng/g for the mutant type (Fig. 8). The lowest concentrations were seen in JA and BR, with the wild type having around 2.6 ng/g and the mutant form having about 1.6 ng/g. The concentration of the wild-type hormone was higher than that of the mutant hormone for each of the six hormones examined. The JA concentration reduced by 28%, BR by 45%, and the other four hormone concentrations decreased in various ratios compared to the wild type in mutant plant buds.

## Discussion

Rapeseed, an important oil crop, is used extensively in the production of biodiesel, food, and other products. Under light simplification and high-density planting, the rapeseed main inflorescence silique yield contributed the most to the yield per plant, and the increase in main inflorescence siliques made it easier to harvest mechanically, thus increasing the level of mechanization. The 16R480 Bnclib gene, which is a derivative of the 12R1402 main inflorescence bud cluster, was mutated. On an average, there were more than 175 main inflorescence siliques, with a maximum of 250. This is appropriate for mechanized production. In this study, two main inflorescence bud cluster materials, 12R1402 and 16R480, as well as two wild-type materials, were used to generate combinations I (12R1402 × Huyou 17) and II (16R480 × 7P71), which were used to conduct reciprocal crosses and observe the offspring of F_1_ and F_2_ to understand the Bnclib inheritance mechanism and gene function.

The segregation ratio between plants carrying Bnclib and wild-type plants in the F_2_ generation was 3:1, according to the F_1_ and F_2_ progeny results. The main inflorescence bud clustering was a single-gene-dominant inheritance, and the Bnclib gene had no cytoplasmic effect. The material segregation ratio of a single plant with the Bnclib and a common type of plant in the F_2_ generation was found to be 3:1 by Chen et al. [26], after analyzing the material of multiple main inflorescence mutants and single main inflorescence common types of *Brassica napus* [27]. This is fundamentally consistent with the findings of this study, showing no cytoplasmic influence and two dominant gene differences between the parents. The segregation ratio between plants carrying Bnclib and wild-type plants in the F_2_ generation was 3:1, according to the F_1_ and F_2_ progeny results. The main inflorescence bud clustering was a single-gene-dominant inheritance, and the Bnclib gene had no cytoplasmic effect. Bnclib revealed that flower buds in the bud-moss stage clustered together in the main inflorescence. The main traits of Bnclib during the fruit-bearing stage include an increase in main branch siliques, the density of main branch knot angles, and many main branches or main branches with forked tops [27]. Paraffin sectioning revealed that some mutant plants had several meristems, the number of flower buds was substantially higher than that in the wild-type, and the structure of the central region of the Bnclib mutant plants was not obvious. The main inflorescence of the mutant plants has several branches because of the ability of the shoot apex to divide, differentiate, and form multiple apical meristems [28]. The FM of mutant plants increased, resulting in flower bud clustering. According to Qi et al. [29], the growth rate of leaf primordia in the rapeseed bud cluster mutant was faster than that in the common type, and several lateral primordia developed in the SAM before progressively dividing into multiple SAMs with a complete structure. The results of this study are consistent with previous studies [30]. In conclusion, Bnclib mutants had single or multiple SAMs. Mutants with a single SAM had more clustered flower buds, which later developed into type I flowers (single main branch tops without bifurcation). Type II (many branches) and type III (single-branch apical bifurcation) plants were generated from the mutant plants with numerous SAM.

The entire genome of combinations I and II of *Brassica napus* was resequenced based on an extreme mixing pool to explore the impact of Bnclib gene mutations on *Brassica napus* materials. SNP and InDel association analyses revealed that Bnclib was located in two association regions on chromosome A03. The two significant association regions on the rape genome were distributed as chrA03 28.30–28.50 Mb and chrA03–random 5.77–5.81 Mb, with 22 and 2 candidate genes, respectively. Owing to its rapidity and accuracy, the BSA analysis approach has been applied in various crop fields [31–34]. Based on the results of BSA analysis, 1292 pairs of SSR primers and 131 pairs of InDel primers were designed, and 27 closely linked molecular markers were screened. Transcriptome analysis of the shoot tips of the Bnclib mutant, wild type, and near-isogenic lines at the budding stage showed that only one gene, BnaA03g53930D, was differentially expressed during the candidate interval. The qPCR results showed that BnaA03g53930D was significantly differentially expressed in the stem tip, which supports the validity of an earlier experiment [35].

The stem ends of the Bnclib near-isogenic line and wild-type Huyou 17 contained considerably different levels of GA, CTK, IAA, SLs, JA, and BR. Six hormones were examined to compare their levels in the mutant and wild-type plants. The content of each hormone was much lower in the mutant than in the wild type, which is consistent with the findings of other researchers [36–39]. Based on earlier investigations, Uddin et al. [40] concluded that the cytochrome P450 protein (CYP90B1) regulates the rate of BR synthesis. BR mutants in *Arabidopsis thaliana* cannot synthesize BR, resulting in a decrease in endogenous BR levels. The mutant plants were dwarfed and exhibited delayed senescence. Based on the observation of the CL-4 phenotype in rice cluster panicles, we hypothesized that CL-4 may be related to BR generation. Cl-4 mutations decrease the amount of endogenous BR, preventing the elongation of rice branches and causing grain clustering. Among the six hormones tested in this study, brassinosterol showed the most significant difference between the mutant and wild-type strains, followed by JA. According to the NCBI, TAIR, and Genoscope databases, BnaA03g53930D is a cytochrome P450 gene that functions similarly to Arabidopsis CYP81D11 in controlling JA synthesis. Further research is required to determine the association between jasmonate, the other five hormones, Bnclib characteristics, and functional verification of BnaA03g53930D.

## Conclusion

*Brassica napus* Bnclib mutant plants can differentiate their shoot tips into many apical meristems, leading to the clustering of flower buds in the main inflorescence. There was no cytoplasmic influence of Bnclib, and clustering of the main inflorescence buds was a single-gene dominant trait. According to the SNP and InDel association analyses, Bnclib was located in the chrA03 28301316–28492,991 and CHRA03-Random 5775817–5806729 association regions of the A03 chromosome, with 22 and 2 candidate genes, respectively. Based on the results of BSA analysis, 27 pairs of primers closely linked to the Bnclib gene were designed and screened. Transcriptomic analysis and qPCR verified that only BnaA03g53930D was differentially expressed in the stem tips of the Bnclib mutant and wild-type plants. Functional annotation indicated that this gene is a cytochrome P450 gene that regulates JA synthesis in *Arabidopsis thaliana*. Further research is necessary on the interactions between JA and the other five hormones, *Brassica napus* main inflorescence bud clustering, and functional verification of BnaA03g53930D.

## Supplementary Information

**Additional file 1: Table S1.** Progeny separation ratio between fascicled main inflorescence mutant lines and wild-type crosses. **Table S2.** Clean Reads, Clean Date, GC content Q20 and Q30 values of R01 and R02 in combination I. **Table S3.** Comparison results of R01, R02 and reference genomes in combination I. **Table S4.** Reads Number, GC content Q20 and Q30 values PL、PM、BM and BL in the combination II. **Table S5.** Comparison results of samples and reference genomes in combination II. **Table S6.** SNP correlation region statistics of F2 population in combination I. **Table S7.** SNP correlation region statistics of F2 population in combination I. **Table S8.** SNP correlation region statistics of F2 population in combination II. **Table S9.** SNP correlation region statistics of F2 population in combination II. **Table S10.** 15 pairs of SSR closely linked primer information. **Table S11.** 12 pairs of InDel closely linked primer information. **Table S12.** Primer information. **Table S13.** PCR amplification. **Supplementary Fig. S1.** qRT-PCR analysis. Differential expression in various organs between Wild-type (Wild) and Mutant-type (Mutant) plants. “*” means significant difference, “**” means extremely significant difference. **Supplementary Fig. S2.** Screening of markers linked to Bnclib and construction of near-isogenic lines. A: Fifteen SSR markers revealed the polymorphisms among two parents and two bulks. B: Twelve InDel markers revealed the polymorphisms among two parents and two bulks. a: 12R1402; b: Huyou 17; c: Bnclib bulk; d: Normal bulk, ns: not selected. **Supplementary Fig. S3.** A: Electrophoretic image of primer chrA03-2. B: Electrophoretic image of primer chrA03-3. C: Electrophoretic image of primer chrA03-6 (Plants No. 1-30 are NIL plants, plants indicated by the arrow are plants with normal bud of main inflorescence). D: Electrophoretic image of primer chrA03-13.P1: 12R1402; P2: Huyou 17;F2-1: Bnclib plant of (12R1402xHuyou 17)F2; F2-1: normal plant of ( 12R1402xHuyou 17)F2.

## Data Availability

All the data related to this study can find on NCBI (https://www.ncbi.nlm.nih.gov/sra/PRJNA962674, Reference ID is = PRJNA962674) and in supplementary files.

## References

[1] Antoniadi I, Plačková L, Simonovik B, Doležal K, Turnbull C, Ljung K, Novák O (2015). Cell-type-specific cytokinin distribution within the Arabidopsis primary root apex. Plant Cell.

[2] Binenbaum J, Weinstain R, Shani E (2018). Gibberellin localization and transport in plants. Trends Plant Sci.

[3] Chen W, Zhang Y, Liu X, Chen B, Tu J, Tingdong F (2007). Detection of QTL for six yield-related traits in oilseed rape (Brassica napus) using DH and immortalized F2 populations. Theor Appl Genet.

[4] Deshmukh UC, Saxena RR, Xalxo MS, Sharma D, Verulkar SB (2013). Hybrid purity testing in rice (Oryza sativa L.) using microsatellite markers. Electron J Plant Breed.

[5] Ding J, Mao LJ, Yuan BF, Feng YQ (2013). A selective pretreatment method for determination of endogenous active brassinosteroids in plant tissues: double layered solid phase extraction combined with boronate affinity polymer monolith microextraction. Plant Methods.

[6] Dong Z, Alam MK, Xie M, Yang L, Liu J, Helal MMU, ..., Liu S. Mapping of a major QTL controlling plant height using a high-density genetic map and QTL-seq methods based on whole-genome resequencing in Brassica napus. G3. 2021;11(7):118. 10.1093/g3journal/jkab118 PMC8495924 33836054

[7] Floková K, Tarkowská D, Miersch O, Strnad M, Wasternack C, Novák O (2014). UHPLC–MS/MS based target profiling of stress-induced phytohormones. Phytochemistry.

[8] Hadfi K, Speth V, Neuhaus G (1998). Auxin-induced developmental patterns in Brassica juncea embryos. Development.

[9] Hu Q, Hua W, Yin Y, Zhang X, Liu L, Shi J, ..., Wang H. Rapeseed research and production in China. Crop J. 2017;5(2):127–135.

[10] Jing L, Chao L, Ruimao Z, Zhineng C, Xianqiang Z, Zhihong G, Pan L. Brassica napus L. dwarfing gene: Determining candidate intervals of dwarfing genes by BSA and SNP typing. bioRxiv. 2020;2020-08.

[11] Khan SU, Yangmiao J, Liu S, Zhang K, Khan MHU. Zhai Y, ..., Zhou Y. Genome-wide association studies in the genetic dissection of ovule number, seed number, and seed weight in Brassica napus L. Ind Crops Prod. 2019;142:111877.

[12] Li B, Zhang X, Liu Z, Wang L, Song L, Liang X, ..., Ma C. Genetic and molecular characterization of a self-compatible Brassica rapa line possessing a new class II S haplotype. Plants. 2021;10(12):2815. 10.3390/plants10122815 PMC8709392 34961286

[13] Li H, Wang Y, Li X, Gao Y, Wang Z, Zhao Y, Wang M (2011). A GA-insensitive dwarf mutant of Brassica napus L correlated with mutation in pyrimidine box in the promoter of GID1. Mol Biol Rep.

[14] Li X, Liu X, Fan Y, Li S, Yu M, Qian M, Lu K. Development of a target capture sequencing SNP genotyping platform for genetic analysis and genomic breeding in rapeseed. Crop J. 2023;11(2);499-510.

[15] Liu G, Zhao T, You X, Jiang J, Li J, Xu X (2019). Molecular mapping of the Cf-10 gene by combining SNP/InDel-index and linkage analysis in tomato (Solanum lycopersicum). BMC Plant Biol.

[16] Liu M, Chang W, Yu M, Fan Y, Shang G, Xu Y, ..., Lu K. Overexpression of defective in Anther Dehiscence 1 increases rapeseed silique length through crosstalk between JA and auxin signaling. Ind Crops Prod. 2021;168:113576.

[17] Lu K, Xiao Z, Jian H, Peng L, Qu C, Fu M, ..., Li J. A combination of genome-wide association and transcriptome analysis reveals candidate genes controlling harvest index-related traits in Brassica napus. Sci Rep. 2016;6(1):1–13. 10.1038/srep36452 PMC5095561 27811979

[18] Manzi M, Gómez-Cadenas A, Arbona V (2015). Rapid and reproducible determination of active gibberellins in citrus tissues by UPLC/ESI-MS/MS. Plant Physiol Biochem.

[19] Nakano H, Takahata K, Mine Y, Sugiyama N (2020). Characterizing the main and epistatic effects and interactions of germination-related quantitative trait loci of tomato using a backcross inbred line population and near-isogenic lines. Sci Hortic.

[20] Qi L, Mao L, Sun C, Pu Y, Fu T, Ma C, ..., Wen J. Interpreting the genetic basis of silique traits in B rassica napus using a joint QTL network. Plant Breed. 2014;133(1):52–60.

[21] Singh VK, Khan AW, Jaganathan D, Thudi M, Roorkiwal M, Takagi H, ..., Varshney RK. QTL‐seq for rapid identification of candidate genes for 100‐seed weight and root/total plant dry weight ratio under rainfed conditions in chickpea. Plant Biotechnol J. 2016;14(11):2110–2119. 10.1111/pbi.12567 PMC5095801 27107184

[22] Song JM, Guan Z, Hu J, Guo C, Yang Z, Wang S, ..., Guo L. Eight high-quality genomes reveal pan-genome architecture and ecotype differentiation of Brassica napus. Natu Plants. 2020;6(1), 34–45. 10.1038/s41477-019-0577-7 PMC6965005 31932676

[23] Song J, Li Z, Liu Z, Guo Y, Qiu LJ (2017). Next-generation sequencing from bulked-segregant analysis accelerates the simultaneous identification of two qualitative genes in soybean. Front Plant Sci.

[24] Song XH, Lei T, Wang SX, Zhou JL, Zhang J, Zan C, ..., Chen YH. Integrating transcriptomic and proteomic analyses of photoperiod-sensitive in near isogenic maize line under long-day conditions. J Integr Agric. 2019;18(6):1211-1221

[25] Sriboon S, Li H, Guo C, Senkhamwong T, Dai C, Liu K (2020). Knock-out of TERMINAL FLOWER 1 genes altered flowering time and plant architecture in Brassica napus. BMC Genet.

[26] Tarkowská D, Novák O, Oklestkova J, Strnad M (2016). The determination of 22 natural brassinosteroids in a minute sample of plant tissue by UHPLC–ESI–MS/MS. Anal Bioanal Chem.

[27] Teixeira RT, Sheng X, Brunner AM (2019). Activity of the shoot apical and cambial meristems: Coordination and responses to environmental signals. Adv Botanical Res..

[28] Uddin MN, Obara M, Yanagihara S, Ishimaru T, Kobayashi N, Fukuta Y (2016). Genetic characterization of introgression lines with the genetic background of the Indica-type rice (Oryza sativa L.) cultivar IR 64 under irrigated lowland and upland conditions. Field Crop Res.

[29] Vallarino JG, Osorio S (2016). Simultaneous determination of plant hormones by GC-TOF-MS. Plant Signal Transduction.

[30] Verslues PE (2017). Rapid quantification of abscisic acid by GC-MS/MS for studies of abiotic stress response. Plant Stress Tolerance.

[31] Wang L, Duan C, Wu D, Guan Y (2014). Quantification of endogenous brassinosteroids in sub-gram plant tissues by in-line matrix solid-phase dispersion–tandem solid phase extraction coupled with high performance liquid chromatography–tandem mass spectrometry. J Chromatogr A.

[32] Wang L, Zou Y, Kaw HY, Wang G, Sun H, Cai L, ..., Li D. Recent developments and emerging trends of mass spectrometric methods in plant hormone analysis: a review. Plant Methods. 2020;16(1):1–17. 10.1186/s13007-020-00595-4 PMC7161177 32322293

[33] Wei QZ, Fu WY, Wang YZ, Qin XD, Wang J, Li J, ..., Chen JF. Rapid identification of fruit length loci in cucumber (Cucumis sativus L.) using next-generation sequencing (NGS)-based QTL analysis. Sci Rep. 2016;6(1):1–11. 10.1038/srep27496 PMC4895147 27271557

[34] Wolko J, Łopatyńska A, Wolko Ł, Bocianowski J, Mikołajczyk K, Liersch A (2022). Identification of SSR Markers Associated with Yield-Related Traits and Heterosis Effect in Winter Oilseed Rape (Brassica Napus L.). Agronomy.

[35] Xiao HM, Cai WJ, Ye TT, Ding J, Feng YQ (2018). Spatio-temporal profiling of abscisic acid, indoleacetic acid and jasmonic acid in single rice seed during seed germination. Anal Chim Acta.

[36] Yuan D, Zhang Y, Wang Z, Qu C, Zhu D, Wan H, Liang Y (2022). BnKAT2 Positively Regulates the Main Inflorescence Length and Silique Number in Brassica napus by Regulating the Auxin and Cytokinin Signaling Pathways. Plants.

[37] Zhang C, Bai MY, Chong K (2014). Brassinosteroid-mediated regulation of agronomic traits in rice. Plant Cell Rep.

[38] Zhao C, Safdar LB, Xie M, Shi M, Dong Z, Yang L, ..., Liu S. Mutation of the Phytoene Desaturase 3 gene causes yellowish-white petals in Brassica napus. Crop J. 2021;9(5):1124–1134.

[39] Zhao Y, Song C, Brummell DA, Qi S, Lin Q, Duan Y (2021). Jasmonic acid treatment alleviates chilling injury in peach fruit by promoting sugar and ethylene metabolism. Food Chem.

[40] Zong-he ZHU, Yong CHENG, Shi-jie MA, Chen HE, Wen-Yin ZHENG, Ke-jin ZHOU, Yuan-shan MA (2016). Genetics of effective silique number on main inflorescence of new germplasm 12R1402 (Brassica napus L.). Chin J Oil Crop Sci.

