# Correction: Mapping and candidate gene analysis of clustered bud on the main inforescence in *Brassica napus* L

**DOI:** 10.1186/s12870-023-04371-z

**Published:** 2023-07-21

**Authors:** Wen Yin Zheng, Zhe Yi Zhu, Abdul Sami, Meng Yuan Sun, Yong Li, Jian Hu, Xing Zhi Qian, Jin Xu Ma, Mei Qi Wang, Yan Yu, Fu Gui Zhang, Ke Jin Zhou, Zong He Zhu

**Affiliations:** 1 College of Agronomy, Anhui Agricultural University, Hefei, China; 2 National Key Laboratory of Wheat and Maize Crop Science, College of Agronomy, Henan Agricultural University, Zhengzhou, China

**Correction:** ***BMC Plant Biol*** **23, 348 (2023)**

**https://doi.org/10.1186/s12870-023-04355-z**

Following publication of the original article [1], an error was identified in the affiliation of author, Abdul Sami. His correct affiliations are Aff1 and Aff2, respectively:

1 College of Agronomy, Anhui Agricultural University, Hefei, China

2 National Key Laboratory of Wheat and Maize Crop Science, College of Agronomy, Henan Agricultural University, Zhengzhou, China

The original article [1] has been corrected.
