# Supplementary material for: Mapping and candidate gene analysis of clustered bud on the main inflorescence in Brassica napus L
Source: BMC Plant Biol. 2023 Jul 4;23:348. doi: 10.1186/s12870-023-04355-z (PMC10318724; doi:10.1186/s12870-023-04355-z)
Supplement: Supplementary file 1 — Additional file 1: Table S1. Progeny separation ratio between fascicled main inflorescence mutant lines and wild-type crosses. Table S2. Clean Reads, Clean Date, GC content Q20 and Q30 values of R01 and R02 in combination I. Table S3. Comparison results of R01, R02 and reference genomes in combination I. Table S4. Reads Number, GC content Q20 and Q30 values PL、PM、BM and BL in the combination II. Table S5. Comparison results of samples and reference genomes in combination II. Table S6. SNP correlation region statistics of F2 population in combination I. Table S7. SNP correlation region statistics of F2 population in combination I. Table S8. SNP correlation region statistics of F2 population in combination II. Table S9. SNP correlation region statistics of F2 population in combination II. Table S10. 15 pairs of SSR closely linked primer information. Table S11. 12 pairs of InDel closely linked primer information. Table S12. Primer information. Table S13. PCR amplification. Supplementary Fig. S1. qRT-PCR analysis. Differential expression in various organs between Wild-type (Wild) and Mutant-type (Mutant) plants. “*” means significant difference, “**” means extremely significant difference. Supplementary Fig. S2. Screening of markers linked to Bnclib and construction of near-isogenic lines. A: Fifteen SSR markers revealed the polymorphisms among two parents and two bulks. B: Twelve InDel markers revealed the polymorphisms among two parents and two bulks. a: 12R1402; b: Huyou 17; c: Bnclib bulk; d: Normal bulk, ns: not selected. Supplementary Fig. S3. A: Electrophoretic image of primer chrA03-2. B: Electrophoretic image of primer chrA03-3. C: Electrophoretic image of primer chrA03-6 (Plants No. 1-30 are NIL plants, plants indicated by the arrow are plants with normal bud of main inflorescence). D: Electrophoretic image of primer chrA03-13.P1: 12R1402; P2: Huyou 17;F2-1: Bnclib plant of (12R1402xHuyou 17)F2; F2-1: normal plant of ( 12R1402xHuyou 17)F2. [file 12870_2023_4355_MOESM1_ESM.docx]

**Results of Bnclib genetic analysis**

**Table S1** Progeny separation ratio between fascicled main inflorescence mutant lines and wild-type crosses

| Group | No. of plants | Cluster main inflorescence | non-clustered main inflorescence | Expected ratio | χ^2^ | *P* |
| --- | --- | --- | --- | --- | --- | --- |
| 12R1402 | 20 | 20 | 0 | - | - | - |
| Huyou 17 | 20 | 0 | 20 | - | - | - |
| 16R480 | 20 | 20 | 0 | - | - | - |
| 7P71 | 20 | 0 | 20 | - | - | - |
| F_1_ (12R1402× Huyou17) | 20 | 20 | 0 | - | - | - |
| F_1_ (Huyou17×12R1402) | 25 | 25 | 0 |  |  |  |
| F_1_ (16R480×7P71) | 20 | 20 | 0 | - | - | - |
| F_1_ (7P71×16R480) | 24 | 24 | 0 |  |  |  |
| F_2_ (12R1402× Huyou17) | 288 | 206 | 82 | 3:1 | 1.6713 | 0.1961 |
| F_2_ (Huyou17×12R1402) | 276 | 215 | 61 | 3:1 | 1.0870 | 0.2971 |
| F_2_ (16R480×7P71) | 243 | 177 | 66 | 3:1 | 0.4952 | 0.4816 |
| F_2_ (7P71×16R480) | 263 | 204 | 59 | 3:1 | 0.7921 | 0.3735 |
| F_1_ (12R1402×Huyou17) ×12R1402(2023) | 100 | 100 | 0 | - | - | - |
| F_1_ (12R1402×Huyou17) ×Huyou17(2023) | 98 | 53 | 45 | 1:1 | 0.5292 | 0.4669 |

**Note:** 12R1402 is clusters bud of main inflorescence type; 16R480 is clusters bud of main inflorescence type; Huyou 17 is wild type; 7P71 is wild type.

**Table S2** Clean Reads, Clean Date, GC content Q20 and Q30 values of R01 and R02 in combination I.

| Samples | Clean Reads | Clean Data | GC Content | %≥Q30 |
| --- | --- | --- | --- | --- |
| R01 | 107410507 | 32223152100 | 38.66 | 93.30 |
| R02 | 89310002 | 26793000600 | 38.26 | 93.43 |

**Table S3** Comparison results of R01, R02 and reference genomes in combination I

| Samples | Number of reads | Mapped | Duplication | Mean mapping quality |
| --- | --- | --- | --- | --- |
| R01 | 218411406 | 98.09 | 29.32 % | 52.772 |
| R02 | 181687730 | 98.80 | 26.94 % | 52.7696 |

**Table S4** Reads Number, GC content Q20 and Q30 values PL、PM、BM and BL in the combination II

| Sample ID | Read Number | BaseNumber | A% | T% | G% | C% | N% | GC% | Q30% | Q20% | Average Q |
| --- | --- | --- | --- | --- | --- | --- | --- | --- | --- | --- | --- |
| PL | 71270625 | 21381187500 | 31.41 | 31.12 | 18.77 | 18.70 | 0 | 37.47 | 89.75 | 96.28 | 35.25 |
| PM | 73663118 | 22098935400 | 31.38 | 31.10 | 18.79 | 18.72 | 0 | 37.51 | 89.13 | 95.99 | 35.13 |
| BM | 121713878 | 36514163400 | 31.36 | 31.10 | 18.8 | 18.73 | 0 | 37.53 | 89.85 | 96.35 | 35.27 |
| BL | 157845788 | 47353736400 | 31.45 | 31.17 | 18.72 | 18.65 | 0 | 37.37 | 89.75 | 96.27 | 35.25 |

**Table S5** Comparison results of samples and reference genomes in combination II

| ID | Total Reads | Mapped Reads | Mapped Paired Reads | Mapped Proper Reads |
| --- | --- | --- | --- | --- |
| BL | 315691576 | 313507175(99.31 %) | 312517600(98.99 %) | 284417972(90.09 %) |
| BM | 243427756 | 241785692(99.33 %) | 241072734(99.03 %) | 218255558(89.66 %) |
| PL | 142541250 | 141696047(99.41 %) | 141287566(99.12 %) | 129565076(90.90 %) |
| PM | 147326236 | 146094217(99.16 %) | 145550582(98.79 %) | 133325078(90.50 %) |

**Bnclib trait gene mapping**

**Table S6,** SNP correlation region statistics of F_2_ population in combination I

| Chr | Starts | End | Size (M) | Genes |
| --- | --- | --- | --- | --- |
| chrA03 | 25377565 | 27580832 | 2.20 | 357 |
| chrA03-random | 4415885 | 4631703 | 0.22 | 12 |
| chrA03-random | 5775817 | 5806729 | 0.03 | 2 |
| chrA09-random | 3397950 | 4017144 | 0.62 | 136 |

**Table S7,** SNP correlation region statistics of F_2_ population in combination I

| Chr | Starts | End | Size (M) | Genes |
| --- | --- | --- | --- | --- |
| chrA03 | 26688888 | 28492991 | 1.8 | 257 |
| chrA09-random | 3311888 | 3886510 | 0.57 | 120 |

**Table S8**, SNP correlation region statistics of F_2_ population in combination II

| Chr | Start | End | Size (M) | Genes |
| --- | --- | --- | --- | --- |
| ChrA03 | 28301316 | 29687267 | 1.39 | 98 |
| ChrA03-random | 2302018 | 6014757 | 3.71 | 151 |
| ChrA04 | 12774567 | 13805054 | 1.03 | 154 |

**Table S9,** SNP correlation region statistics of F_2_ population in combination II

| Method | Chr | Start | End | Size (M) | Genes |
| --- | --- | --- | --- | --- | --- |
| InDel-index | chrA03-random | 4978732 | 5751914 | 0.77 | 31 |

**Screening of markers linked to Bnclib and construction of near-isogenic lines**

**Table S10**, 15 pairs of SSR closely linked primer information

| No. |  | Forward primer（5'-3'） | Reverse primer（3'-5'） |
| --- | --- | --- | --- |
| 1 | chrA03-1 | CGGAGATGAGGAAGCTTTTG | TACTGCTCCCAAACCTGGAC |
| 2 | chrA03-2 | AGGGGGAGAGCGAGATAGAG | ATGAAAATGGTTGCAAAGGC |
| 3 | chrA03-3 | TTTGCTGATTGATTTTTGTTGG | CTCCCGACGTGTTTGGTTAT |
| 4 | chrA03-4 | CACGGGTTCTGGAGATGATT | ATGGTCCATCTAAACGTGGC |
| 5 | chrA03-5 | AACTGGGGCAAGATTGAATG | GGAAGGCAGCTCAAGAAAGA |
| 6 | chrA03-6 | ACCGTTGGGCTTTTCTTTCT | GCTTGAAGGCTTTTGGAGTG |
| 7 | chrA03-7 | AGTCGAATCACGTCCCAAAG | TTCCCATGTGGTCACGTAAG |
| 8 | chrA03-8 | TCCATTCTTCCTTTGGCTTG | TTTGAAGCGAGTCTTCAGCA |
| 9 | chrA03-9 | CAGGTGGTCTTTGTGTTCTCA | GGAAGGCCATTGATACAAAGTT |
| 10 | chrA03-10 | TTGCAGAGACGAGACAGCAG | GGTCCCATCCCTTCATCTCT |
| 11 | chrA03-11 | ACGACATGGTCAATGCGTAA | CGTGGATTCACATCGTTCAC |
| 12 | chrA03-12 | TCTGCCCTCCTCTCCATCTA | GGCTGATATGGGCTTTTGTC |
| 13 | chrA03-13 | TCAGCAACAGAAGCAGAGGA | GTGTTTGATGCAGACTTGCG |
| 14 | chrA03-14 | GAGTTTTTGGGAGTTGGTGC | TTCGGATCTGATCTGGGAAC |
| 15 | chrA03-15 | TGACCAGGTTTTTAGATCGCA | TTTCAGCGCTTGGAGAGAAT |

**Table S11,** 12 pairs of InDel closely linked primer information

| No. |  | Forward primer（5'-3'） | Reverse primer（3'-5'） |
| --- | --- | --- | --- |
| 1 | chrA03-16 | GAACGATGTAGATCAAAGAAACCA | TCAGAGTTGACAACAGCAACAA |
| 2 | chrA03-17 | AAAAGCTGTTTATTCCCGGC | GTCGCACTCTGTCCTCCTTC |
| 3 | chrA03-18 | ATGTGGGATGGCGAGATATT | GTGTGCCAAAACAGAGATGC |
| 4 | chrA03-19 | GTGAACCATGGTGAGACCAA | CCATGATGATTGATGGGTGA |
| 5 | chrA03-20 | GGCTTGCATTTACCAACACA | AATCTCAAAGCGAGAAAGCG |
| 6 | chrA03-21 | GCCCGGTGCTTTCTACTTTA | TTCAGTTCCACAAAAACCCA |
| 7 | chrA03-22 | TCGCTCTCCTTCTCACTTCC | CGCCATTGTGGATAAACTCA |
| 8 | chrA03-23 | GCGGTGAGACACAACCTCTT | CAGTTGAGCGCACTACATCAA |
| 9 | chrA03-24 | CGCCGTTTTACGTGATTTTT | TGATGACCCAAGAAAGGAACA |
| 10 | chrA03-25 | GGGAGCAATTGGATCTCTGA | CGCCATTCATCTCTTTCCAT |
| 11 | chrA03-26 | TGTATTATTCCTGTTCCCAAATATAAA | AAACAATCAAAAATTCACATGTTTC |
| 12 | chrA03-27 | AGAACGGATTTAGCAACCGA | ACGTTAATTTTCAAGGCGGT |

**Table S12**, Primer information

| No. |  | Forward primer（5'-3'） | Reverse primer（3'-5'） |
| --- | --- | --- | --- |
| 1 | chrA03-2 | AGGGGGAGAGCGAGATAGAG | ATGAAAATGGTTGCAAAGGC |
| 2 | chrA03-3 | TTTGCTGATTGATTTTTGTTGG | CTCCCGACGTGTTTGGTTAT |
| 3 | chrA03-6 | ACCGTTGGGCTTTTCTTTCT | GCTTGAAGGCTTTTGGAGTG |
| 4 | chrA03-13 | TCAGCAACAGAAGCAGAGGA | GTGTTTGATGCAGACTTGCG |

**Table S13, PCR amplification**

| Segment | Number of cycle | Temperature (℃) | Duration (s) |
| --- | --- | --- | --- |
| 1 | 1 | 94 | 300 |
| 2 | 10 | 94 | 60 |
|  |  | 60 | 30 |
|  |  | 72 | 45 |
| 3 | 30 | 94 | 60 |
|  |  | 55 | 30 |
|  |  | 72 | 45 |
| 4 | 1 | 72 | 720 |
| 5 | 1 | 4 | ∞ |

**Supplementary Fig. S1. qRT-PCR analysis**
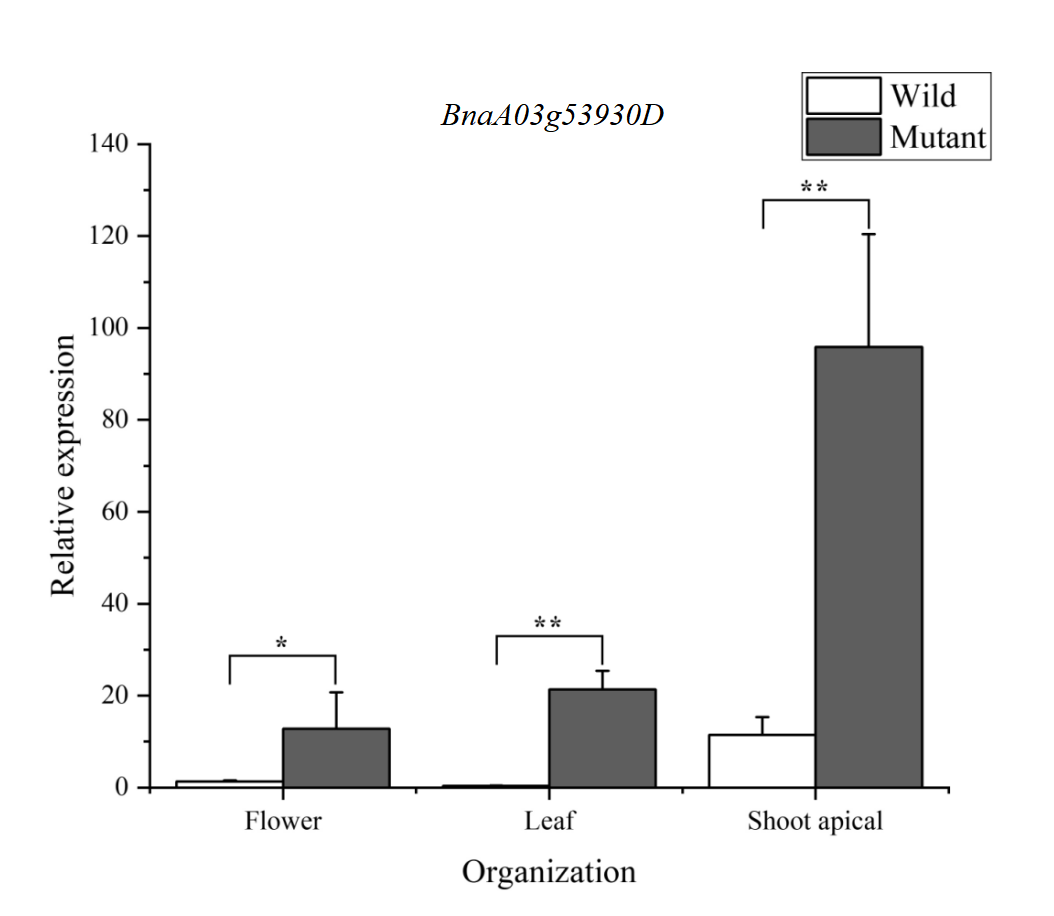


**Supplementary Fig. S1,** differential expression in various organs between Wild-type (Wild) and Mutant-type (Mutant) plants. “*” means significant difference, “**” means extremely significant difference.

**Supplementary Fig. S2 Screening of markers linked to Bnclib and construction of near-isogenic lines**


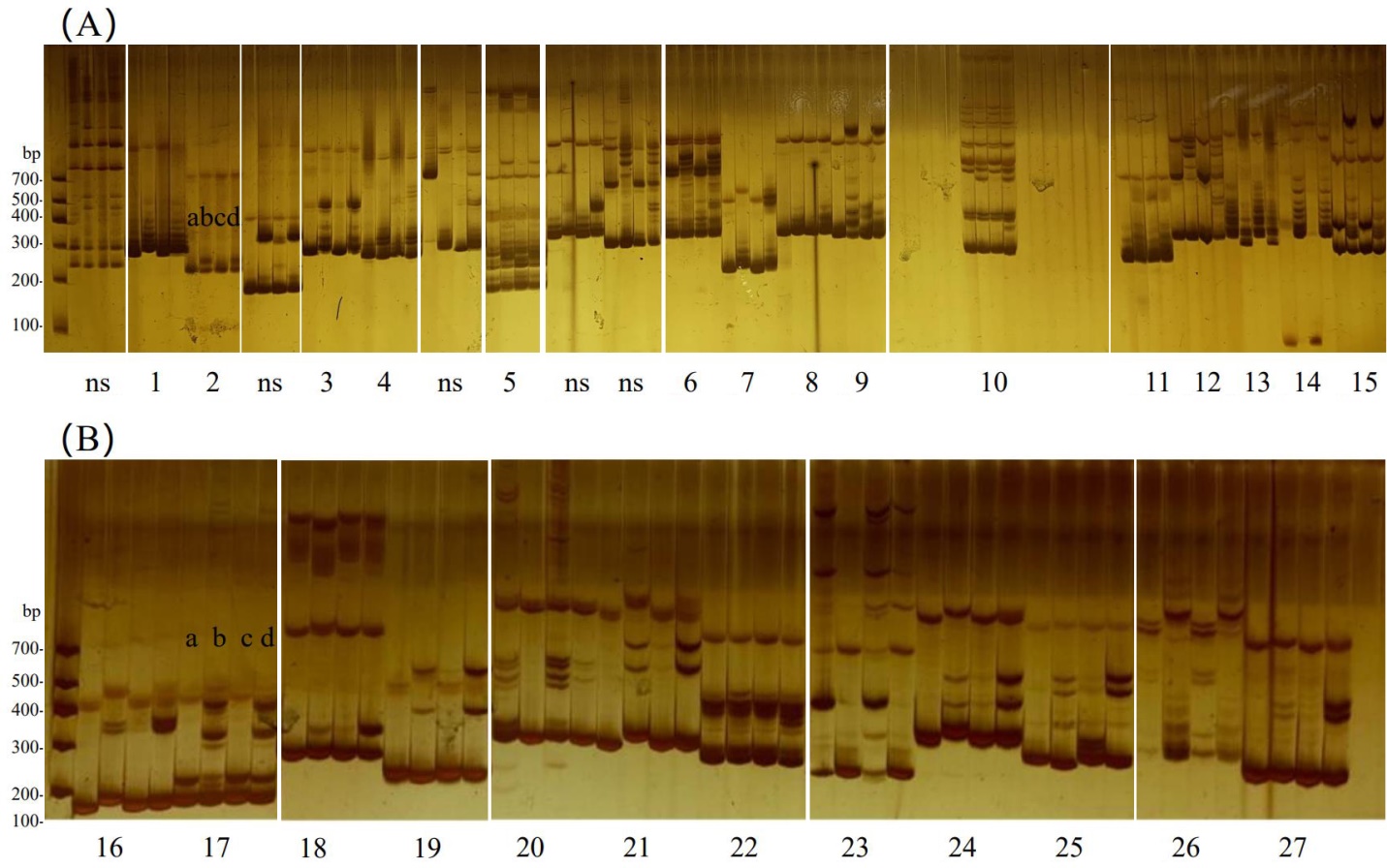


**Supplementary Fig. S2. A:** Fifteen SSR markers revealed the polymorphisms among two parents and two bulks. **B:** Twelve InDel markers revealed the polymorphisms among two parents and two bulks. **a**: 12R1402; **b**: Huyou 17; **c**: *Bnclib* bulk; **d**: Normal bulk, ns: not selected

**Supplementary Fig. S3.**


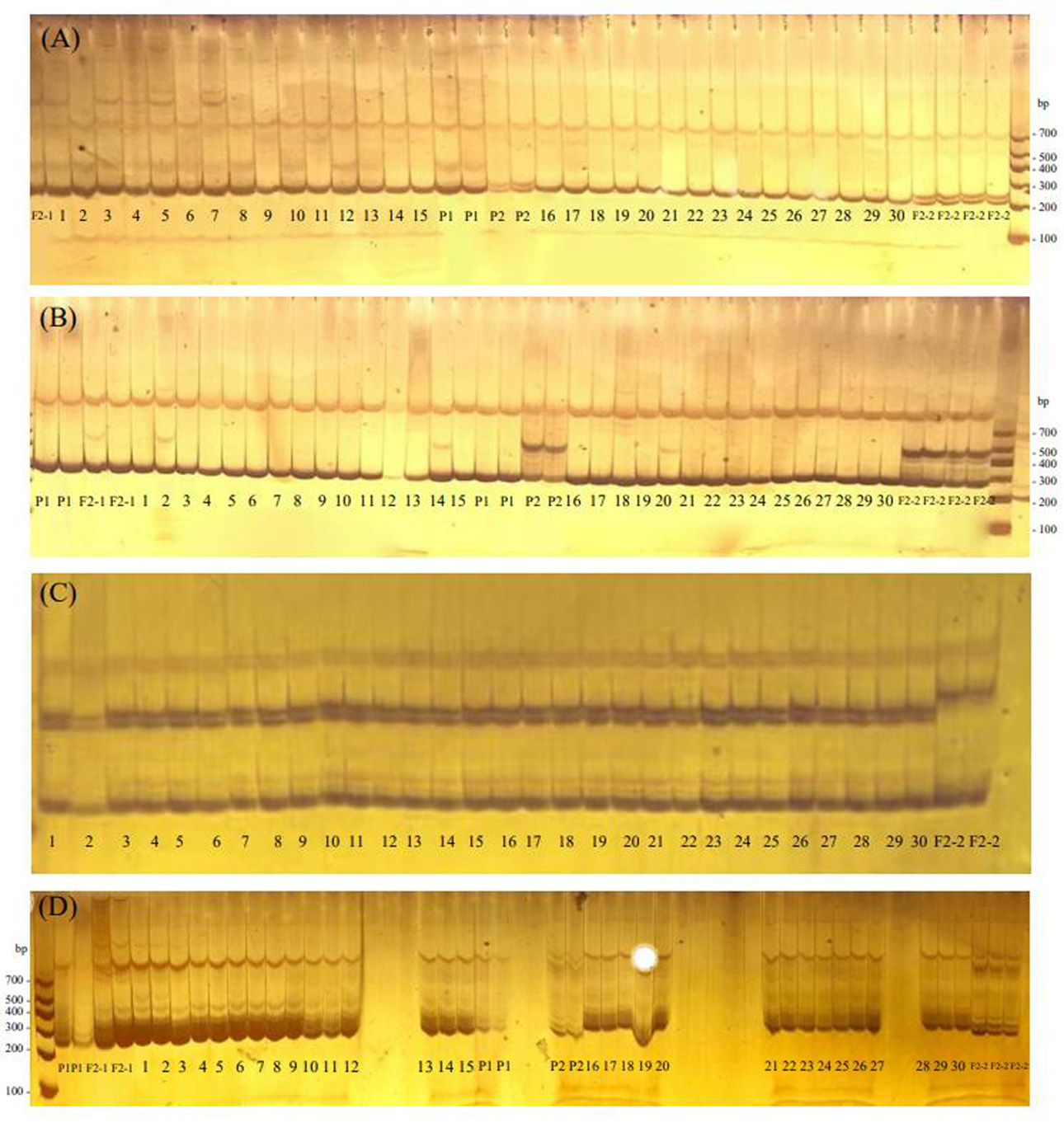


**Supplementary Fig. S3. A:** Electrophoretic image of primer chrA03-2. **B:** Electrophoretic image of primer chrA03-3. **C:** Electrophoretic image of primer chrA03-6 (Plants No. 1-30 are NIL plants, plants indicated by the arrow are plants with normal bud of main inflorescence). **D:** Electrophoretic image of primer chrA03-13.P_1_: 12R1402; P_2_: Huyou 17;F_2_-1: *Bnclib* plant of ( 12R1402ⅹHuyou 17)F_2_; F_2_-1: normal plant of ( 12R1402ⅹHuyou 17)F_2_
